# Staff's perspectives on physical activity in acute mental health general adult wards

**DOI:** 10.1192/bjo.2021.545

**Published:** 2021-06-18

**Authors:** Nikoletta Lekka, Samantha Nunns, Robert Verity

**Affiliations:** Sheffield Health and Social Care NHS Foundation Trust

## Abstract

**Aims:**

Physical activity (PA) has multiple health benefits for people with severe mental illness (SMI). Nevertheless, people with SMI engage in less exercise and more sedentary behaviour than the general population. Additionally, inpatient settings can exacerbate barriers to PA and facilitate sedentary behaviour. Staff's attitudes towards PA promotion may influence patient engagement. The aim of this study was to explore staff's views on PA for acute psychiatric inpatients, including enablers and barriers.

**Method:**

An online anonymous survey with free text was sent to all 85 multidisciplinary team (MDT) members of two acute general adult wards, including nurses, doctors and allied health professionals. A qualitative approach was used to gain deeper understanding of the participants' perspectives. Manual thematic analysis was completed to identify discrete themes.

**Result:**

Response rate was 64%, with 54 professionals responding. Notably, 100% agreed or strongly agreed that exercise is beneficial to physical and mental health. Nevertheless, 72% felt it was not easy to do PA with patients during their shift, while many reported they were able to encourage exercise but were unable to accompany patients to sessions. Specifically, participants reported lack of time (40%), high level of clinical activity (32%), lack of staff (30%), lack of PA resources inside the wards (20%) and conflicting priorities (18%), stopping them from helping patients to do more exercise. However, they felt more staff (28%), time dedicated to PA (26%), on-ward resources (18%), access to the gym and gardens (18%), staff dedicated to PA (16%) and staff trained in facilitating PA (10%), would help participants promote PA on the ward. Other suggestions to enable PA included a change in ward culture, valuing and promoting PA, daily patient encouragement by all MDT members instead of only occupational therapists, and PA promotion as part of mental health treatment and as physical health strategy. Finally, 70% of participants said they exercised regularly, although some reported lack of time or motivation, work commitments and workload-related exhaustion reducing their ability to exercise.

**Conclusion:**

Participants acknowledged the importance of PA for physical and mental health. Furthermore, they described multiple enablers and barriers. Prioritising PA during admission, providing on-ward activities, educating/training staff, reiterating that PA promotion is within all MDT members' job roles, and offering organisational support can contribute to improved PA provision and regular involvement of patients.An integrative approach to mental health and wellbeing, promoting PA in inpatient psychiatric settings is required.

